# Cooling down is as important as warming up for a large-bodied tropical reptile

**DOI:** 10.1098/rspb.2024.1804

**Published:** 2024-11-06

**Authors:** Kaitlin E. Barham, Ross G. Dwyer, Celine H. Frere, Lily K. Bentley, Cameron J. Baker, Hamish A. Campbell, Terri R. Irwin, Craig E. Franklin

**Affiliations:** ^1^ School of the Environment, University of Queensland, Saint Lucia, Queensland 4072, Australia; ^2^ School of Science, Technology and Engineering, University of the Sunshine Coast, Maroochydore, Queensland 4556, Australia; ^3^ Centre for Biodiversity and Conservation Science, University of Queensland, Saint Lucia, Queensland 4072, Australia; ^4^ Research Institute for Environment and Livelihoods, Charles Darwin University, Darwin, Northern Territory 0909, Australia; ^5^ Australia Zoo, Steve Irwin Way, Beerwah, Queensland 4519, Australia

**Keywords:** thermal type, thermoregulation, basking, intra-individual variation, acoustic telemetry

## Abstract

An ectotherm’s performance and physiological function are strongly tied to environmental temperature, and many ectotherms thermoregulate behaviourally to reach optimum body temperatures. Tropical ectotherms are already living in environments matching their thermal tolerance range and may be expected to conform to environmental temperatures. We tracked the body temperatures (*T*_b_) of 163 estuarine crocodiles across 13 years and compared *T*_b_ of 39 crocodiles to water temperature gathered using fish-borne sensors (*T*_w_) across 3 years (2015–2018). While *T*_b_ largely conformed closely to *T*_w_, we found inter- and intra-individual differences in relative body temperature (*T*_b_–*T*_w_) that depended on sex and body size as well as the time of day and year. Deviations from *T*_w_, especially during the warm parts of the year, suggest that thermoregulatory behaviour was taking place: we found patterns of warming and cooling events that seemed to mediate this variation in *T*_b_. Thermoregulatory behaviour was observed most frequently in larger individuals, with warming events common during winter and cooling events common during summer. By observing free-ranging animals across multiple years, we found that estuarine crocodiles show yearly patterns of active cooling and warming behaviours that modify their body temperature, highlighting their resilience in the face of recent climate warming. Our work also provides the first evidence for thermal type in large-bodied reptiles.

## 1. Introduction

The rate and effectiveness of biological functions are directly influenced by body temperature, and so changes in temperature can affect fitness. Ectotherms depend on external sources of heat, and so their body temperature and physiological performance are strongly linked to environmental temperatures [1,2]. To mitigate the potential negative impacts of unfavourable environmental temperatures, individuals should seek to maintain their body temperature within their optimum range either by acclimatizing (reversibly adjusting their thermal sensitivity) to environmental temperatures or by thermoregulating behaviourally [3]. Behavioural thermoregulation allows ectothermic animals to select favourable thermal microclimates, and many studies in temperate environments have demonstrated that small-bodied reptile species will bask to reach thermal optima that are higher or lower than ambient environmental conditions [4–7]. These species can often maintain relatively stable body temperatures by taking advantage of warming or cooling sources in their environment [7,8].

In tropical latitudes, reptiles frequently live in thermally stable environments with ambient temperatures equal to or greater than their optimal range [9,10]. As such, it has been hypothesized that tropical species can maintain relatively stable body temperatures without the need for behavioural thermoregulation by allowing their body temperature to conform to ambient temperatures (i.e. thermoconformity) [11–13]. Even so, the selection of cool microclimates is often necessary to prevent overheating during the heat of the tropical summer [12,14,15]. As these organisms are likely already living at the higher end of their thermal tolerance range, and due to constraints on biochemical processes, they may have a limited capacity to adapt to warmer temperatures within the time frame of anthropogenically induced climate change [9,10,16], which is predicted to result in warmer average temperatures and additional climate variabilities such as extreme weather events [17].

Recent studies have begun to document that body temperature may vary among individuals in a population (i.e. inter-individual variation in body temperature), with some individuals consistently seeking warmer or cooler temperatures than their conspecifics across spatial and temporal contexts. These differences may be related to intrinsic differences such as sex or body size [11,18]. Female ectotherms, for instance, have been shown to seek warmer temperatures, particularly around the breeding season, to provide the energy required to reproduce [11]. Larger individuals have also been shown to exhibit greater thermal inertia and tend to cool down more slowly [18]. Even social status has been shown to influence thermoregulatory behaviour through the exclusion of subordinates from basking spots [4,19]; thus, the body temperature of some individuals should vary more than others (i.e. intra-individual variation in body temperature). These differences may also be influenced by personality (consistent individual differences in a trait). While the personality of classic traits such as activity and boldness is well established [20–22], the personality of thermal type and thermoregulatory behaviour is only beginning to be studied [21,23–26]. If individuals in a population do maintain consistent differences in their body temperature, then they must do this by thermoregulating towards their target temperature across geographical and seasonal shifts in environmental temperatures. While much research into thermoregulatory behaviour has been conducted on small, temperate ectotherms [13], less is known for larger, long-lived species in tropical latitudes. Furthermore, it is often difficult to account for inter- and intra-individual variation in thermal type due to the logistical difficulties of tracking individuals and their surrounding thermal environment at the spatial and temporal scale or grain at which they interact with the environment [27].

**Figure 1.**
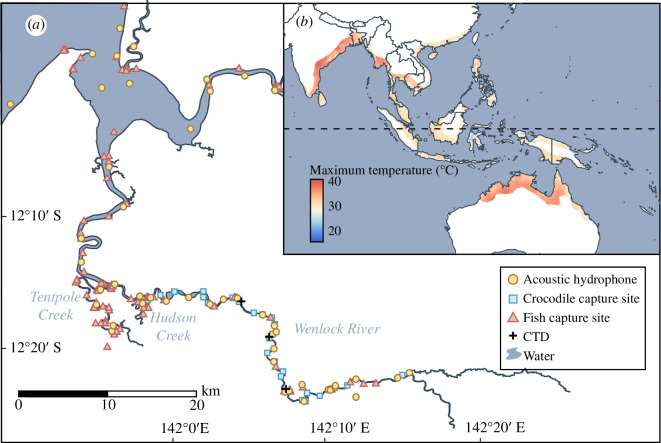
(*a*) The acoustic array on the Wenlock and Ducie Rivers, Cape York, Queensland, Australia, showing the locations of the acoustic receivers as yellow circles, crocodile capture sites as blue squares and fish capture sights as red triangles. Conductivity, temperature and depth loggers (CTDs) are represented by black crosses. (*b*) Maximum temperatures reached across the global distribution of estuarine crocodiles *C. porosus*. Temperature data come from WorldClim [28] and represent the maximum yearly temperature reached from 1970 to 2000, at a resolution of one pixel per 170 km^2^.

Estuarine crocodiles *Crocodylus porosus* are large, long-lived ectotherms that are widely distributed around the equator (figure 1). They have been observed shifting between land and water to buffer against daily and seasonal temperature fluctuation [19,29]. Juveniles in laboratory conditions have been shown to maintain performance across a broad variety of temperatures through thermal acclimation [30] and have demonstrated a substantial ability to maintain their aerobic scope under climate change-like temperatures (34°C) through acclimation [31]. However, they show reductions in both swimming and diving performance above 32–33°C, a temperature that is commonly exceeded across their range [30,32,33]. Estuarine crocodiles undergo a 20-fold increase in body size over their lifetime, and so mature animals are expected to interact differently with their thermal environment. However, due to the difficulty in recording the body temperature of mature crocodiles in the wild, where they are exposed to natural temperatures and temperature fluctuations, their range of inter- and intra-individual variation in thermal type has not been investigated.

In this study, we aimed to quantify long-term variations in body temperature and observe patterns of thermoregulatory behaviours in wild estuarine crocodiles. To achieve this, we collected 13 years of body temperature data (*T*_b_) from 163 wild estuarine crocodiles surgically implanted with temperature-sensitive acoustic transmitters. To measure water temperature (*T*_w_) at the same spatial and temporal scale as the tagged crocodiles, we also utilized acoustically tagged sharks, rays and bony fishes (52 individuals; five species) as animal-borne temperature sensors [27,34]. Our aims were as follows: (i) investigate to what degree individuals conformed to *T*_w_ by analysing inter- and intra-individual variation in *T*_b_ relative to *T*_w_, (ii) investigate whether patterns of warming or cooling indicative of thermoregulation are present, and (iii) determine whether thermoconformity or thermoregulation change through time or with an individual’s body size or sex. We hypothesized that large males would have the warmest body temperatures, due to their high thermal inertia and high social rank allowing them preferential access to basking areas. Additionally, we hypothesized that basking would be most frequent during the winter months, while crocodiles would seek to remain cool during the summer.

## 2. Methods

### (a) Study site, crocodile capture and sampling

This study was conducted in the Wenlock River, Cape York, Australia (figure 1). This region experiences a warm wet season from November to April (25–39°C) and a cooler dry season from May to October (20–34°C). Between 2008 and 2021, up to 20 crocodile traps were deployed along a 47 km stretch of the Wenlock River. Traps were set between August and September each year and either floated on the water surface or were placed at the high-tide mark along the riverbank. Traps were baited with wild pig (*Sus scrofa*) or cow (*Bos taurus*), with the trap door sprung by a trigger mechanism attached to the bait. For individuals less than 2 m in total length, hand capture via spotlighting with a lasso was also used.

### (b) Remote monitoring of crocodile temperature

To track the body temperature (*T*_b_) of individual crocodiles remotely across multiple years (max = 10 years), coded acoustic transmitters (V13T or V16T; https://www.innovasea.com/) were implanted into captured crocodiles following Franklin *et al*. [35]. These acoustic transmitters contain inbuilt temperature sensors that allow the remote monitoring of crocodile presence and temperature over multiple years (max = 10 years). In brief, a local anaesthetic (lignocaine with adrenaline) was injected behind the left forelimb, and a small pocket formed under the skin with blunt-ended scissors, into which the acoustic transmitter was inserted and placed on top of skeletal muscle. The incision was closed using monofilament sutures and sprayed with antibiotic. As these sensors were implanted close to muscle, they may not accurately reflect the core body temperature of very large individuals with a greater internal thermal gradient but rather a value between the core body temperature and local environmental temperatures. While crocodiles were restrained, sex, total body length (TL), snout–vent length (SVL) and tail girth were also recorded before individuals were released at their point of capture.

To detect the implanted transmitters, an array of acoustic receivers (VR2-W; https://www.innovasea.com/) was deployed throughout the Wenlock River for the duration of the study. These receivers were spaced approximately 1–5 km apart and were attached to concrete anchors positioned approximately 1–2 m below the water surface and 2–20 m from the riverbank. Each receiver had a detection radius of 200–700 m. As the river width was typically less than 100 m and pulse transmission rate of the acoustic tags was set randomly between 90 and 120 s, it was unlikely that crocodiles could pass by a receiver without being detected.

Individuals with fewer than 30 days of detections, or fewer than 100 detections overall, were excluded, as were erroneous readings (for instance, less than 10°C). Some data from 15 individuals, as well as all data from three individuals, were removed due to rapid, progressive drops in temperature to 0°C that were not replicated in either the water temperature or the *T*_b_ of other tagged crocodiles at the time.

### (c) Environmental temperature data

Local water temperatures represent the operative temperature for exclusively aquatic ectotherms [36], and so we obtained approximate water temperature from coded acoustic transmitters (V13T) implanted into free-swimming fish and sharks (barramundi *Lates calcarifer* (*n* = 10, 0.45–1.06 m total length), bull sharks *Carcharhinus leucas* (*n* = 15, 0.78–1.18 m total length), speartooth sharks *Glyphis glyphis* (*n* = 8, 0.64–1.43 m total length), estuarine whipray *Urogymnus dalyensis* (*n* = 7, 0.49–1.13 m total length) and warrior catfish *Hemiarius dioctes* (*n* = 12, 0.49–1.04 m total length)) detected on the same array of acoustic receivers [37,38].

As almost all fish are obligate thermoconformers [39], and the flowing and tidal Wenlock River lacks a significant thermal gradient (electronic supplementary material, figure S1), these readings were accurate to the actual water temperature of the river, as derived from three instream temperature recorders (Star-Oddi conductivity, salinity and depth loggers (CTDs)) which were in place from October 2015 to 2016 (figure 1). Individuals within fish species conformed to water temperatures and did not show individual specialization in thermal type (electronic supplementary material, figure S2). All fish temperature recordings were within 3.1°C of CTD recordings made within the same 3 h period and within 3 km of each other, and 90% of fish recordings were within 1.1°C of CTD recordings. We used fish body temperature (hereafter *T*_w_) to approximate water temperature for years when there was sufficient data (2015–2018, more than 100 000 recordings per year), and erroneous readings were excluded as with the crocodiles.

To calculate the relative temperature of crocodiles to their environment (*T*_r_), we matched the mean *T*_b_ of each crocodile within a fixed 3 h window to the mean *T*_w_ recorded at the same acoustic receivers as the focal crocodile within the same window. This 3 h window was chosen to allow within-day variation in *T*_r_ to be observed, while maximizing the overlap between *T*_b_ and *T*_w_ recordings. *T*_r_ was then calculated as *T*_b_–*T*_w_.

### (d) Inter- and intra-individual variation and plasticity of crocodile temperature

All statistical analyses were conducted using the R statistical software v. 4.3.1 [40]. We constructed a double-hierarchical generalized linear model (DHGLM) following the methods of Hertel *et al.* [41] and using the ‘brms’ R package [42], to determine whether there were inter-individual differences (behavioural type (BT)) or intra-individual differences (residual intra-individual variation (rIIV)) in *T*_r_. We also estimated the correlation between BT and rIIV. The DHGLM was run with uninformative priors. Four chains were run for a total of 8000 iterations, of which 6000 comprised the warm-up period. The ‘mean model’ component of the DHGLM consisted of *T*_r_ as a response variable. Month was included as a nonlinear second-order polynomial term to account for the cyclical fluctuation of temperature through the year, while time of day (grouped in 3 h windows) was included as a factor to account for the irregular shape of the diurnal cyclical pattern of temperature. Crocodile body size or sex was included as a fixed effect with four levels comprising females (*n* = 12, SVL 410–1648 mm), small males (*n* = 9, SVL < 1470 mm), medium males (*n* = 9, 1470 mm ≤ SVL < 1700 mm) and large males (*n* = 9, 1700 mm ≤ SVL < 2510 mm). The size categories for males match with size at maturity (approx. 1500 mm SVL; [43]) and with known shifts in movement strategy [44]. Random intercepts for both individual ID and month within study year were included. The ‘dispersion model’ component of the DHGLM consisted of the standard deviation of *T*_r_ fitted against a random intercept for individual ID, with body size or sex included as a fixed effect.

The repeatability of *T*_r_ was calculated as $R=\frac{IDvar}{TOTALvar}$, where IDvar is inter-individual variation, and TOTALvar is the sum of IDvar, between-year variation, month-within-year variation and residual variation. The predictability of *T*_r_ was estimated using the coefficient of predictability (CVp), calculated as $CV_{P}=\sqrt{\exp\left(\omega_{ID}^{2}\right)-1}$, where *ω*_ID_ is the estimate of individual differences in residual variation. After BT and rIIV were extracted, rIIV was back transformed to the original scale by adding population-level mean residual variance and taking its exponent.

### (e) Identification of warming and cooling periods

**Figure 2.**
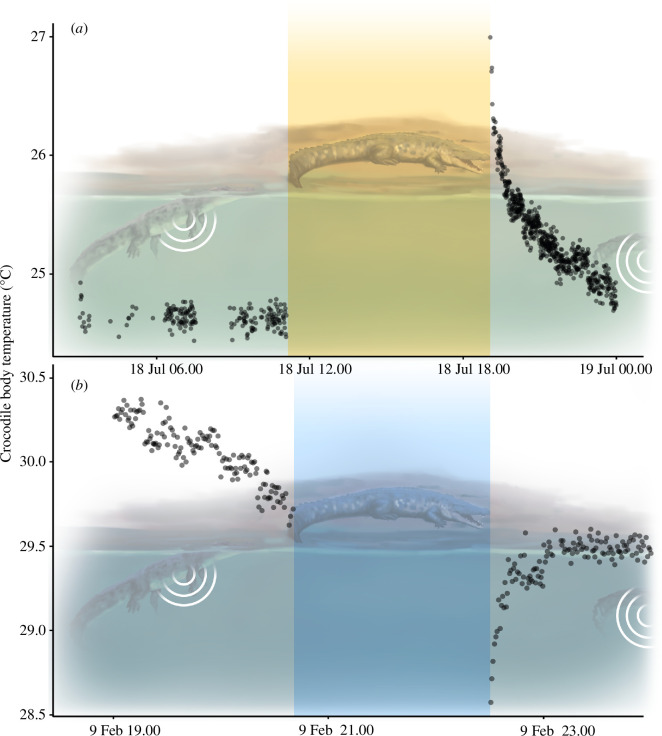
Temperature recordings from two estuarine crocodiles *C. porosus* showing (***a***) a warming event (tag ID 3076) and (***b***) a cooling event (tag ID 3093). The space between detections (yellow and blue rectangles) likely represented a period where the crocodiles were out of the water basking or actively cooling, as they were unable to be detected during this time.

We expect that crocodiles that never leave the water will largely conform to water temperature, although this will be influenced by thermal inertia related to body size. However, crocodiles are known to behaviourally modify their body temperature by leaving the water and either warming in the sun or cooling evaporatively in the shade [19]. Individuals that are out of the water (e.g. on the riverbank or mudflat) are unable to be detected by the acoustic array until they return to the water. To identify potential periods of thermoregulation, we used the R package ‘VTrack’ [45] to search for instances in our dataset (2008−2022; 163 individuals) when a tagged crocodile was not detected for periods ranging between 30 min and 24 h and had a *T*_b_ that was either warmer or cooler by a threshold value (1.3°C) than before this interval (figure 2). This threshold was calculated as the 99% highest residual *T*_r_. The total number of events per individual per month was then calculated. We used the R package ‘mgcv’ [46] to build four generalized additive models, two each for warming and cooling events, and males and females, to visualize the relationship between events and time of year. The months of February–April were not included in this analysis due to a low number of crocodile detections associated with wet season flooding. The number of basking events per month, fitted using a Poisson distribution, was the response variable, with month of the year and body size as continuous predictors fitted using a tensor product spline with *k* set between 4 and 9 and individual ID as a random effect.

## 3. Results

**Figure 3.**
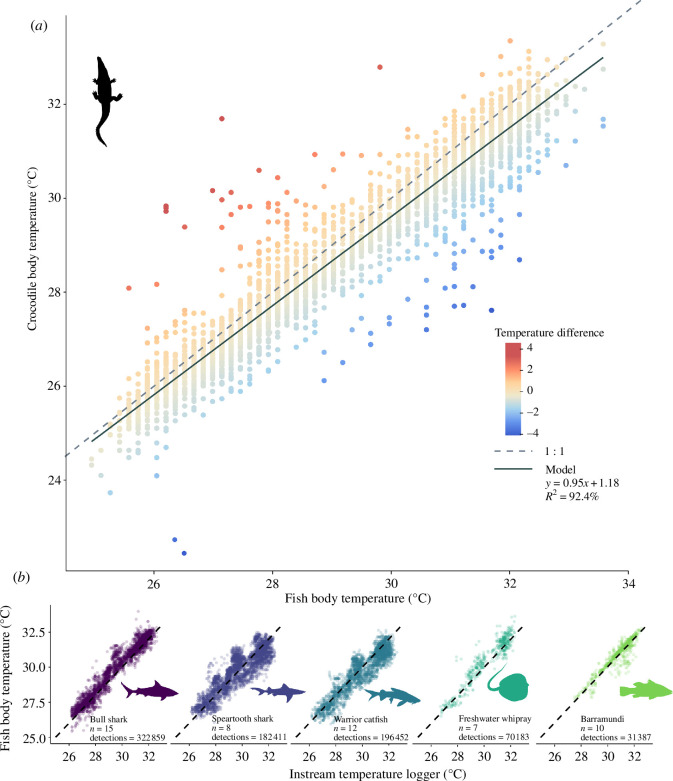
(***a***) Scatter plot showing the relationship between the body temperature of 39 estuarine crocodiles *C. porosus* and their immediate water temperature as determined using fish-borne temperature sensors. Crocodile and fish temperatures are joined by a shared location within a 3 h window. (***b***) The relationship between tag temperatures for five fish species and the nearest instream temperature logger. The dashed lines show a 1 : 1 relationship between temperature values.

Between August 2008 and September 2022, 223 crocodiles (0.56–4.69 m TL) were captured on the Wenlock River and implanted with acoustic tracking devices. Of these, 163 individuals (0.84–4.67 m TL) had sufficient data (body temperature recorded at least 100 times over at least 30 days) to be included in the analysis for warming and cooling events, and 39 individuals (0.84–4.64 m TL) could be matched with fish temperature data gathered concurrently between 2015 and 2018 to be used in the analysis of thermal type. Crocodile *T*_b_ was found to match closely to *T*_w_ values, and both *T*_b_ and *T*_w_ followed cyclical seasonal trends, with the warmest temperatures occurring in January and the coolest temperatures in July. However, there was more variation in *T*_b_ than in *T*_w_ (figure 3); *T*_b_ ranged from 21.0 to 39.9°C whereas *T*_w_ ranged from 24.0 to 34.8°C, and crocodile body temperature varied by up to 4.55°C warmer or 4.08°C cooler than the local water temperature.

### (a) Inter- and intra-individual variation and plasticity of body temperature

**Figure 4.**
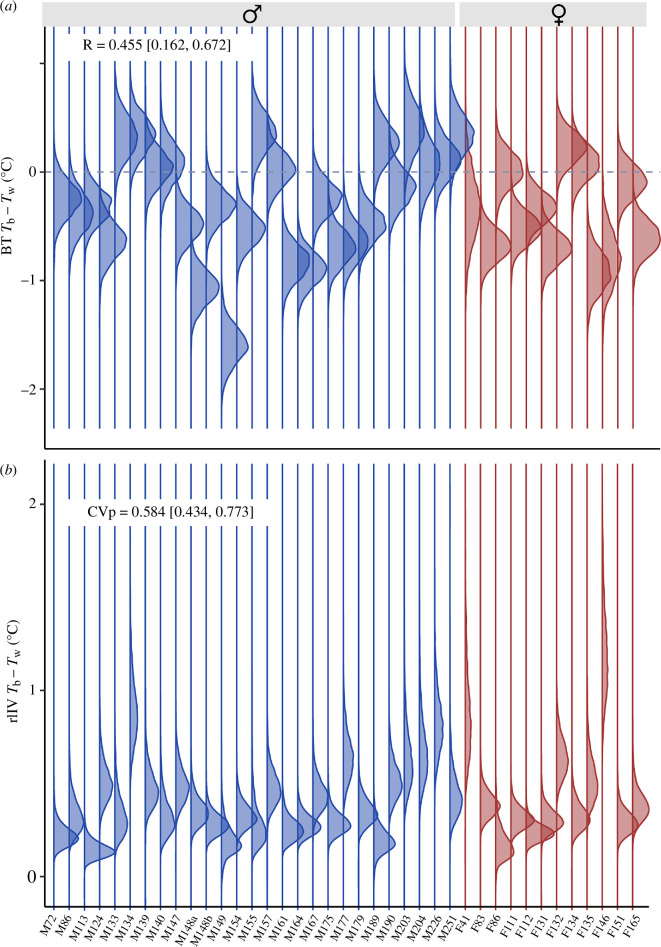
Distributions of inter- and intra-individual variation in body temperature relative to water temperature of 39 estuarine crocodiles *C. porosus*. (*a*) Behavioural type of estuarine crocodiles, such that individuals below the dotted line are on average cooler than the water, while those above are warmer. (*b*) Residual intra-individual variation of estuarine crocodiles, such that individuals closer to 0 are more consistent. Individuals are coloured by sex and sorted according to sex and body size where M134 is a male 1340 mm SVL.

Crocodiles were, on average, 0.34°C cooler than the local water temperature. As this was less than the 0.5°C temperature sensor accuracy reported by the acoustic tag manufacturers (Innovasea), this variation may simply represent transmitter error. In one crocodile dual-tagged with two acoustic transmitters between September 2010 and April 2012, 100% of co-detections occurring within a 30 min period were within 0.42°C, and 90% were within 0.17°C, suggesting a greater degree of accuracy. The coolest individual in our dataset was on average (±standard error) 1.33 ± 0.05°C cooler than the water, and the warmest individual was on average 0.47 ± 0.18°C warmer than the water (figure 4).

**Figure 5.**
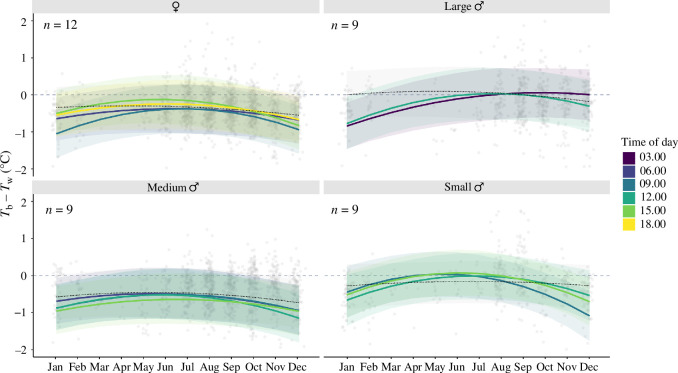
Body temperature (*T*_b_) of estuarine crocodiles *C. porosus* (*n* = 39) relative to the water temperature (*T*_w_) throughout the year. Crocodiles are grouped by body size, with females, large males (greater than 1700 mm SVL), medium males (1470–1700 mm SVL) and small males (less than 1470 mm SVL). Coloured lines show times of day when the slope of the relationship between temperature and time of year is nonlinear or different from 0. The black dotted lines show the relationship between temperature and time of year for other times of day, while the grey dashed line shows when *T*_b_ = *T*_w_. Grey dots are jittered raw data.

We found distinct inter-individual differences in *T*_r_, with 46% of variation in *T*_r_ attributed to individual differences (*R* = 0.455 ± (0.162, 0.672); figure 4). A three-way interaction between body size, time of year and time of day was supported (figure 5). When controlling for time of year and time of day, large males (greater than 1700 mm SVL) were the warmest group, and medium males (1470–1700 mm SVL) were the coolest. Crocodiles were never warmer than the water, but females, small males (less than 1470 mm SVL) and large males were generally cooler than the water during the mornings of hotter months (figure 5*a,b,d*; see electronic supplementary material, table S1 for estimates and credible intervals of each combination of size or sex, time of day and time of year). Medium male *T*_r_ was less than zero for most of the year, except for during the late night (figure 5*c*).

The mean residual standard deviation in *T*_r_ was 0.38°C, and there was significant intra-individual variation in *T*_r_ (CVp: 0.584 [0.434, 0.773]; figure 5*b*), though an individual’s degree of variation was not influenced by its sex or body size and did not correlate with its *T*_r_ (table 1).

**Table 1.** Estimates and 95% credible intervals of random effects of estuarine crocodile *C. porosus* body temperature relative to local water temperature (°C) throughout the year, based on a double hierarchical mixed model. Bolded estimates have credible intervals that do not overlap 0.

random effects	estimate
s.d._intercept,crocodile ID_	**0.48 [0.37, 0.62]**
ω^2^_crocodile ID_	**0.54 [0.40, 0.72]**
*r*_intercept(crocodile ID)–ω(crocodile ID)_	0.06 [−0.31, 0.43]
s.d._intercept,year_	**0.40 [0.12, 1.24]**
s.d._intercept,year : month_	**0.06 [0.04, 0.09]**

### (b) Warming and cooling events

Warming or cooling events were defined as instances when tagged crocodiles were not detected for a period of 30 min to 24 h and became warmer or cooler by a threshold value following this absence. On average, crocodiles were not detected for 7.6 h (7.5 h for cooling or 7.9 h for warming) and once detected, returned to within 1°C of their original body temperature in 1.9 h (1.5 h post-cooling or 2.3 h post-warming). A total of 3033 ‘warming’ and 3710 ‘cooling’ events were recorded across 163 crocodiles from 2010 to 2022, with a mean of 3.05 cooling events per individual per month and 2.50 warming events per individual per month. The greatest number of warming (*n* = 19) events were recorded from a large male crocodile (Tag ID = 11897; 2510 mm SVL) in July of 2019, and the greatest number of cooling (*n* = 46) events was recorded from a small male (Tag ID = 3133; 1250 mm SVL) in December of 2021. This individual was restricted to a waterhole for much of the year, and so was picked up consistently by the acoustic receiver at this location. Cooling events were most common in the early morning, peaking at 06.00, while warming events were most common in the late afternoon, peaking at 19.00. As these events represent the timing of a potential return to the water, the crocodile may have been basking for hours previously.

**Figure 6.**
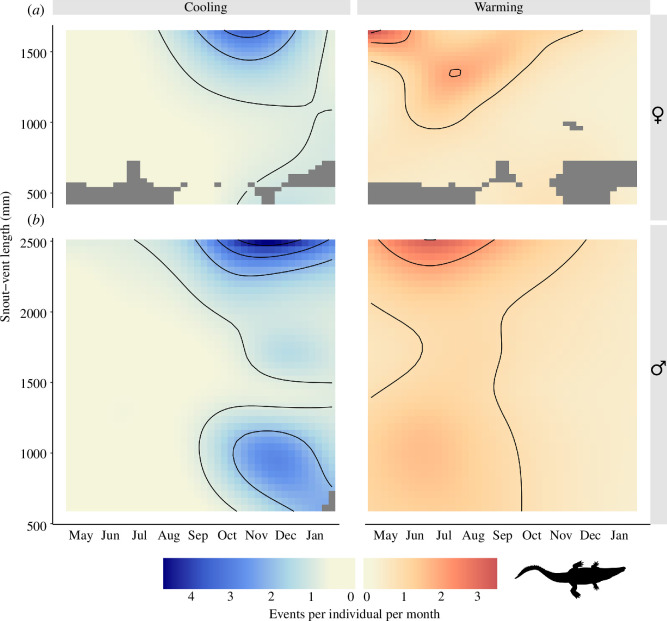
Contour plots showing the frequency of cooling and warming events of (***a***) 55 female and (***b***) 108 male estuarine crocodiles *C. porosus* through the year. Colours represent the modelled mean number of cooling or warming events per individual per month, with grey rectangles representing a lack of data.

The smoothing parameter for the interaction between body size and time of year was significant for all four models (cooling/female: edf = 10.42, *Χ*^2^ = 410.24, *p* < 0.001; warming/female: edf = 20.85, *Χ*^2^ = 164.70, *p* < 0.001; cooling/male: edf = 24.55, *Χ*^2^ = 524.07, *p* < 0.001; warming/male: edf = 16.83, *Χ*^2^ = 178.57, *p* < 0.001). Warming events were most common in July for both females (figure 6*a*) and males (figure 6*b*). Very large males (greater than 2000 mm SVL) warmed the most frequently, and males with 1000–2000 mm SVL the least. Conversely, basking was positively correlated with SVL in females. Cooling events were most common in November for females and December for males. Similarly, males with greater than 2000 mm SVL had the most frequent cooling events among this cohort, while intermediate-sized males had the fewest cooling events. For females, cooling was most common for individuals greater than 1200 mm SVL.

## 4. Discussion

While ectotherms from the thermally stable tropics are likely to be living near their upper thermal limits, thermoregulation may provide a buffer against unfavourable temperatures. By tracking the body temperatures of a population of wild estuarine crocodiles over multiple years and using fish-borne temperature loggers to characterize local environmental conditions, we found that tagged crocodiles mostly conformed to local water temperature but that their relative body temperature depended on their sex and body size, as well as the time of day and time of year. Crocodiles of all sizes were often cooler than the water in summer; periods of time when body temperature increased or decreased substantially relative to water temperature, suggestive of behavioural thermoregulation, may have mediated this variability in temperature. Crocodiles were able to behaviourally thermoregulate to cool down in summer, as well as warm up in winter, and thermoregulatory behaviour was size-dependent, with a greater frequency of both warming and cooling observed for larger individuals and very small individuals. These results suggest that, while estuarine crocodiles mainly conform to water temperatures, they do have some capacity to elevate or lower their body temperature at need.

While most obligate aquatic ectotherms must conform to local water temperatures [47–49], semi-aquatic ectotherms may thermoregulate behaviourally by leaving the water to take advantage of solar radiation, evaporative cooling or ambient air temperature to assist them in reaching a target body temperature [3,50,51]. We found that crocodiles mostly conformed to water temperature, but we observed individual differences in *T*_r_ (body temperature relative to environment temperature) that ranged from 1.33°C cooler to 0.47°C warmer than the water. There was also significant intra-individual variation in *T*_r_, though an individual’s degree of variation did not correlate with its *T*_r_ and was not influenced by its sex or body size. In contrast, Horváth *et al*. [23] found that the thermal type (selected temperature) of common lizards *Zootoca vivipara* was negatively correlated with its intra-individual variance, such that individuals selecting for higher temperatures were more predictable. The largest crocodiles (i.e. males greater than 1700 mm SVL) tended to be warmest, and medium-sized males (1475–1700 mm SVL) tended to be the coolest. This parabolic pattern of behaviour has also been observed in the diet [52] and movement [44] of male estuarine crocodiles, with medium-sized males tending to be more nomadic and consuming prey of a higher trophic level than both immature and large dominant males. Rather than reflecting a preference for warmer temperatures, the warmer body temperature of large male estuarine crocodiles may simply be a consequence of their size: at upward of 500 kg, these individuals have considerable thermal inertia, and their body temperature changes slowly. Grigg *et al*. [19] found that an estuarine crocodile over 5.5 m in total length maintained stable temperatures at a daily scale, though not at a yearly scale. However, our largest (greater than 1700 mm SVL males) crocodiles had a similar degree of intra-individual variation in *T*_r_ than medium-sized males. This may be because none of our tagged crocodiles were above approximately 4.6 m in total length (largest 4.64 m TL) and due to differences in our methodologies. While Grigg *et al*. [19] measured core body temperature with ingested radio transmitters, our acoustic transmitters were implanted close to the surface (approx. 8–12 mm) and measured peripheral body temperature, so may have experienced greater temperature fluctuations. A difference of 1–2°C has been observed between the core and peripheral temperatures of mature American alligators, *Alligator mississippiensis* [53].

Individual differences in thermal preference have been well documented for several species of lizard and fish in a laboratory setting [21,24,54–56]. For ectotherms with the mass, and therefore thermal inertia, of crocodilians, selecting for and maintaining preferred temperatures through thermoregulation may be impractical. Laboratory studies of thermal preference often involve an artificial temperature gradient [21], but there was little observed change in water temperature with either depth or upstream extent in our Wenlock River study system (electronic supplementary material, figure S1), and so our population of estuarine crocodiles lacks a significant temperature gradient to navigate while submerged. Rather, individuals conformed to water temperatures, though *T*_r_ depended on body size or sex, the time of year and the time of day, such that individuals were cooler than the water on summer mornings or midday and matched water temperature the rest of the time. These deviations of *T*_r_ may be linked to behavioural thermoregulation, though individuals that were warmer did not necessarily bask to warm up more than others (electronic supplementary material, figure S3). It is possible that additional factors are contributing towards the differences observed. If these temperature differences are capturing distinct thermal preferences rather than intrinsic factors, these differences may also be linked to personality type. Because of the central role temperature plays for ectotherms, integrating thermal type and thermoregulatory behaviour into the study of ectotherm personality is an area of growing interest. A thermal-behavioural syndrome, linking thermal preference, thermoregulatory behaviour and personality traits such as boldness and activity, has been demonstrated for the delicate skink *Lampropholis delicata* [24,25]. Within this syndrome, individuals who preferred warmer temperatures were often more bold, explorative and active than others [24,25]. A similar pattern of warmer individuals tending to be more active and bold was found in common lizards, with bolder individuals theoretically more inclined to bask (warm) in the face of predator risk [23]. While this is difficult to show in the field, the development of animal-borne sensors with accelerometer, temperature and pressure sensing technologies could be used to investigate the relationship between thermal preferences and activity or diving behaviour in wild crocodiles.

For large ectotherms such as estuarine crocodiles, thermoregulation may be ineffective for reaching a target temperature. Adult female northern map turtles *Graptemys geographica* were unable to reach the same peak temperatures while basking as adult males, due to their significantly larger size and greater thermal inertia, resulting in a decreased effect of thermoregulation [57]. Crocodilians may instead seek to raise or lower their body temperature in the short term, for example, to aid in digestion [58], to optimize performance upon their return to water and to buffer against environmental extremes. Seebacher *et al*. [59] found that temperate-living alligators were mostly thermoconformers, relying on acclimatization to compensate for temperature changes, but were nonetheless warmer than expected in winter and colder than expected in summer. We observed more cooling events in summer, and more warming events were in winter, which may explain why tagged crocodiles were slightly cooler than water temperatures during the morning and middle of the day in summer but otherwise tended to conform to water temperature. Using focal observations of 11 estuarine crocodiles in a naturalistic setting, Grigg *et al*. [19] found that crocodiles spent most time out of the water on summer nights (when the air was cooler than the water) and during winter days (when solar radiation provided warmth). Our study suggests that these behaviours may be common in wild estuarine crocodiles. Although behavioural thermoregulation may offer a physiological advantage to ectotherms, basking can reduce the time available for important behaviours that require submersion (e.g. feeding, territory defence and reproduction) and can expose individuals to agonistic interactions with conspecifics [60]. Dominant male crocodiles have been observed harassing smaller mature males away from basking spots, resulting in these smaller males having the most variable body temperatures [19,29]. While we found medium males were frequently cooler than large, presumably dominant males, their intra-individual variance in body temperature was not greater. Conversely, dominant male Australian water dragons *Intellagama lesueurii* bask less due to time spent defending their territories [4]. We found a general trend of larger crocodiles (both male and female) basking more frequently than medium crocodiles, though warming and cooling events were also frequently recorded for immature males.

While it is likely that the periods of warming and cooling observed are representative of behavioural thermoregulation, the existence of thermoregulation cannot be verified without the associated behaviours being observed. The use of basking behaviour to warm up is well established in crocodilians [3,5,19,59], and in our own study, estuarine crocodiles on the Wenlock River were often observed basking in the sun on riverbanks and mudflats during the cooler July and August. Cooling behaviour is more difficult to observe because it may take place underwater, away from the river proper or at night. The ability to cool behaviourally or access cool refugia is essential for tropical ectotherms exposed to high temperatures [14,15,61]. Some fish species have been observed to take advantage of layers of warmer and cooler water in order to thermoregulate [56,62]. Male tropical fiddler crabs *Austruca mjoebergi* rely on shaded microclimates to remain active throughout the day [61], while mountain lizards *Eurolophosaurus nanuzae* use the cool wind to prevent overheating [7]. Nocturnal basking has recently been described in freshwater turtles and has been linked to species occurring in tropical and subtropical regions and to warmer parts of the year [63,64]. The Wenlock River is shallow for most of the year (max depth = approx. 10 m) and does not appear to be thermally stratified (electronic supplementary material, figure S1). As such, it seems unlikely that crocodiles were taking advantage of deeper water to cool down. Instead, they may have been cooling evaporatively [65], lying in the shade or in cool freshwater runoff from local springs or basking at night. Our crocodiles were detected more frequently after a cooling event at 06.00 and after warming at 19.00, so nocturnal basking may be a primary mechanism of behavioural cooling. Future work linking the verified presence of nocturnal basking to drops in body temperature is required to determine exactly how and when estuarine crocodiles cool down.

To conclude, by using a long-term dataset of estuarine crocodile body temperature, we found that estuarine crocodiles typically conformed to water temperatures but employed both warming and cooling behaviours throughout the year. The existence of cooling behaviour means that crocodiles may have the means to compensate behaviourally for increases in temperature associated with climate change, alongside physiological compensation. We also found consistent inter- and intra-individual differences in body temperature, which may have been mediated through thermoregulatory behaviour or through body size and sex. This contributes toward the growing field of thermal personality and provides the first evidence of individual differences in thermal type in a large-bodied reptile. These findings support the idea that thermoregulation plays a less important role for crocodilians than for other reptiles and that basking to warm up is an equal priority to cooling down for tropical ectotherms.

## Data Availability

Data and code are available on UQ eSpace [66], and data are available on the Acoustic Animal Tracking Database (https://animaltracking.aodn.org.au) of the Integrated Marine Observing System (IMOS, www.imos.org.au). IMOS is a national collaborative research infrastructure supported by the Australian Government. Supplementary material is available online [67].
